# Five-Year Real-World Outcomes of Hymenoptera Venom Immunotherapy: Clinical Effectiveness and Immunological Modifications

**DOI:** 10.3390/toxins18040187

**Published:** 2026-04-15

**Authors:** Claudia Panzera, Sebastiano Gangemi, Luisa Ricciardi

**Affiliations:** Allergy and Clinical Immunology Unit of G. Martino Hospital, Department of Clinical and Experimental Medicine, University of Messina, 98124 Messina, Italy; claudiapanzera07@gmail.com (C.P.); gangemis@unime.it (S.G.)

**Keywords:** Hymenoptera venom allergy (HVA), anaphylaxis, venom immunotherapy (VIT), Vespula, Apis, *Vespa crabro*, *Polistes dominula*, venom-specific IgE, tryptase

## Abstract

Hymenoptera venom allergy is a cause of anaphylaxis, which significantly affects patients’ daily lives due to the constant fear of accidental stings. Venom immunotherapy (VIT) is the only treatment capable of preventing severe systemic reactions (SSRs). Limited long-term real-life data are available, integrating both clinical and immunological outcomes. A five-year prospective observational study was conducted on 35 patients with a history of SSR who underwent VIT at a tertiary allergy center in Southern Italy; two of them had a diagnosis of systemic mastocytosis. Most patients were sensitized to Vespula, but others to Apis, *Polistes dominula* and *Vespa crabro*, reflecting the exposure pattern characteristic of Mediterranean regions. Clinical outcomes following accidental re-stings and serological trends, including total IgE, venom-specific IgE, and baseline serum tryptase, were assessed at treatment initiation and after five years of maintenance therapy. During the entire follow-up, all patients tolerated VIT. No SSRs occurred after accidental stings in 17/35 patients, confirming clinical protection achieved with VIT. Vespula serum-specific IgE presented a highly significant decrease; total IgE, tryptase and specific IgE for Apis, *Polistes dominula* and *Vespa crabro* showed a statistically significant decrease. Our findings reinforce the role of VIT as a well-tolerated, effective and disease-modifying treatment in a real-world setting.

## 1. Introduction

### 1.1. Background

Hymenoptera venom allergy (HVA) is a leading cause of anaphylaxis in adults and represents a major public health concern due to its unpredictable clinical course and potential for life-threatening reactions [1]. Systemic reactions (SRs) after bee or wasp stings may rapidly involve multiple organ systems, occasionally progressing to fatal outcomes [2]. The prevalence of HVA varies widely across Europe and is strongly influenced by climatic and ecological factors, with the highest rates observed in Mediterranean regions [3] with exposure to wasps such as *Vespula* and *Polistes dominula* and *Vespa crabro* [4,5]. Following a severe sting reaction, after the acute episode, many patients experience persistent anxiety, behavioral avoidance, and impaired quality of life [6].

### 1.2. Risk Factors

Several clinical and biological variables increase the risk of severe systemic reactions (SSRs); male sex, outdoor occupations, and outdoor environmental exposure are well-established epidemiological risk factors [7,8].

Baseline serum tryptase (BST) has emerged as a crucial biomarker as elevated levels thereof correlate with clonal mast cell activation syndromes (MCASs) [9] or confirmed systemic mastocytosis (SM), predisposing to SSR after Hymenoptera stings [10].

### 1.3. Diagnosis of HVA

The diagnosis of HVA requires an integrated approach combining clinical history, possible identification of the culprit insect by the patient, and demonstration of venom sensitization [11]. Skin prick tests and intradermal tests can be performed; however, their sensitivity may be temporarily reduced shortly after a sting; a “refractory period” during which skin testing may yield false-negative results, potentially delaying accurate diagnosis, has been reported [12]. Furthermore, guidelines suggest that skin tests may be omitted if a clear result is obtained from laboratory in vitro tests [13]. Therefore, serum venom-specific IgE (s-IgE) is a key diagnostic element [14].

### 1.4. HVA Treatment

HVA can manifest in Hymenoptera venom-sensitized subjects with either a mild, moderate or severe life-threatening reaction. Treatment includes emergency treatment and de-sensitization with VIT.

Emergency treatment for cutaneous large local reactions are oral antihistamines, such as Cetirizine 10 mg, and topical steroid creams; the only available intramuscular H1-antagonist is chlorpheniramine 10 mg, which is useful for systemic allergic reactions to Hymenoptera stings together with intramuscular (IM) or intravenous (IV) steroids such as Hydrocortisone or Methylprednisolone 500 mg, up to 1000 mg according to vital signs and symptomatology of the patient after a reaction. Epinephrine i.m. 0.3 mL and IV fluids should also be administered, if available. During the acute phase, transportation to the Emergency Department is advisable. Epinephrine auto-injectors should be prescribed to patients with confirmed HVA. Epinephrine auto-injections are safe and can be lifesaving if a Hymenoptera sting SR develops. Patients with IM, obesity, or living/working far from Emergency Departments can be prescribed two epinephrine auto-injectors; a second epinephrine injection can be lifesaving if the patient has not completely recovered from anaphylaxis after the first auto-injection. A self-management plan for anaphylaxis should also be provided to HVA patients [15].

Hymenoptera venom immunotherapy (VIT) is the only disease-modifying treatment for HVA and is highly effective in preventing SR upon re-sting, reducing the risk to less than 5% in appropriately treated patients [16].

VIT clinical efficacy is a consequence of pathogenetic immunological effects, including suppression of venom s-IgE, induction of IgG4 “blocking antibodies,” expansion of regulatory T cells, and modulation of mast cell and basophil activation; these changes contribute to immune tolerance to Hymenoptera stings [17].

### 1.5. Study Rationale

VIT in high-risk populations sensitized to Hymenoptera venom is essential to induce protection from occasional stings, as robust evidence supports VIT as an effective treatment preventing fatal reactions in patients under treatment [18]. No reactions or mild reactions are reported with vespid VIT, between 91% and 96%, while between 77% and 84% with bee VIT [19]. Real-life data that integrate both clinical outcomes, such as tolerance to occasional stings, and immunological parameters are still limited. In a study on 70 patients sensitized to *Apis mellifera*, treated for ≥5 years with either aqueous or depot VIT extracts, it was reported that one-half of patients from each group were re-stung during the study, and all suffered only minor discomfort, and that the reduction in serum-specific IgE was significant [20].

It has been suggested that VIT should be performed at least for 5 years, as the risk of recurrence of clinical allergy to stings after VIT is higher in patients who have received VIT for less than 5 years [21].

The present study aimed to provide real-world evidence on the efficacy of VIT in patients with severe HVA, followed during a five-year VIT treatment cycle, evaluating the tolerance to occasional Hymenoptera stings, as well as the variation in immunological parameters such as total IgE levels, Hymenoptera venom s-IgE and BST levels were analyzed at T0, before starting VIT treatment, and at T1 after 5 years of VIT.

### 1.6. Data Statistical Evaluation

To evaluate data collected during the study descriptive and inferential statistical methods were applied, expressing continuous variables as median values and categorical variables as absolute numbers and percentages. At the end of the study baseline and follow-up values were compared to assess temporal variations in immunological parameters. 

## 2. Results

### 2.1. Patients’ Demographic and Clinical Characteristics

The study population included 35 HVA patients, 29 males (82.9%) and 6 females (17.1%), with a mean age of 45.3 years, who had previously experienced, after a Hymenoptera sting, an SSR classified according to Mueller’s classification [22]; 20 patients (57.1%) reported grade IV reactions, characterized by cardiovascular involvement and/or loss of consciousness, while 15 patients (42.9%) experienced grade III reactions, mainly involving respiratory symptoms. A diagnosis of SM was present in two patients with a grade IV reaction after a Hymenoptera sting.

### 2.2. Identification of the Culprit Insect

As the sting challenge cannot be performed in Italy for ethical reasons, we showed the patient an entomological notice board to facilitate the identification of the stinging insect [19]. It has been reported that 73% of Vespula-allergic patients accurately identify this kind of Hymenoptera on the board [23] and that 62.6% of patients recognized the stinging insect, 30.1% as a honeybee and 32.5% as a wasp [24]. The stinging insect in our population was, therefore, recognized in most patients. Vespula was recognized as the culprit insect in 24/25 patients, *Vespa crabro* in 4/4 patients, and Apis in 6/6 patients, respectively. Only one patient was unable to identify the stinging insect.

### 2.3. Venom Sensitization Profile and VIT Extract

Sensitization to vespids’ venom within the cohort of patients was predominant. Vespula sensitization was identified in 25/35 patients. Co-sensitization to *Polistes dominula* was present in 20/25 patients sensitized to Vespula. As Vespula had been recognized as the stinging Hymenoptera causing the SSR, and Vespula s-IgE was observed to be higher than *Polistes dominula* serum s-IgE, all Vespula-sensitized patients, even if co-sensitized to *Polistes dominula*, were treated with Alutard Vespula VIT. Mono-sensitization to Apis was observed in 6/35 patients, who were treated with Alutard Apis VIT, while mono-sensitization to *Vespa crabro* was observed in 4/35 patients, who were treated with Anallergo *Vespa crabro* VIT.

### 2.4. VIT Clinical Outcomes

During the 5-year VIT treatment, 18 patients did not experience any accidental re-stings, while 17 patients reported at least one re-sting event. Clinical manifestations were markedly attenuated compared to the grade of reaction experienced before starting VIT, either grade 0 or grade 1 according to Mueller’s classification. Specifically, 5 patients developed only local reactions (grade 0), 11 patients experienced large local reactions (grade 1), and 1 patient reported after re-sting both local and large local reactions on different occasions. No grade II, III or IV reactions occurred after a Hymenoptera occasional re-sting during VIT maintenance treatment, as no SR was recorded even in patients with previous grade IV reactions and those affected by SM. VIT was well tolerated by all patients. No severe adverse event was recorded during either the build-up or maintenance phases. Overall, VIT showed a favorable safety profile in this real-world cohort.

### 2.5. Laboratory Immunological Parameters

**Figure 1:**
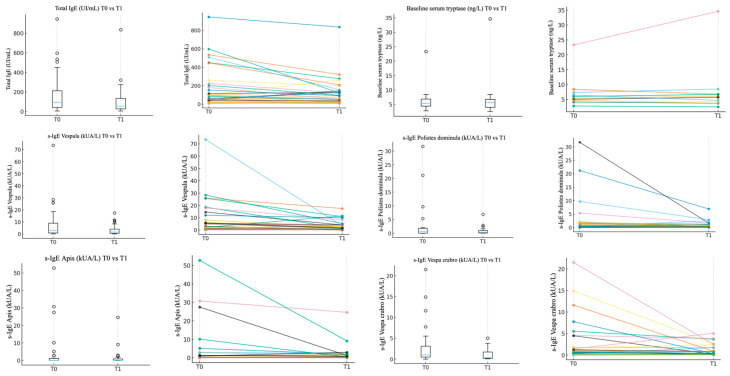
The graphics show the decrease in Total IgE, SBT levels, Vespula s-IgE, *Polistes dominula*-IgE, Apis s-IgE and *Vespa crabro* s-IgE from T0 before VIT treatment to T1 after 5-year treatment. The different colored lines correspond to individual patients.

After five years of VIT treatment, total IgE and Hymenoptera venom s-IgE decreased from T0 to T1. Vespula s-IgE decrease was highly statistically significant (*p* 0.0003); *Vespa crabro* (*p* 0.0222) and Apis (*p* 0.0133) s-IgE levels, as well as total IgE (*p* 0.0028) levels, decreased statistically significantly. In patients sensitized to both Vespula and *Polistes dominula*, and treated with Alutard Vespula, *Polistes dominula* s-IgE also decreased statistically significantly (*p* 0.0177). BST levels also showed a reduction over the five-years VIT treatment, with either Alutard Vespula, Alutard Apis or Anallergo *Vespa crabro* VIT (median Δ of 0.5 μg/L), but not statistically significant (*p* = 0.49). The results obtained from serological investigations at the beginning of VIT treatment (T0) and after five years (T1) are shown in Table 1 and Figure 1. Detailed patient-level immunological data are reported in Supplementary Table S1.

## 3. Discussion

The real-world nature of this study on HVA patients undergoing VIT treatment for a 5-year period allowed for the assessment of both immunological and clinical outcomes beyond a controlled experimental setting. VIT in our five-year follow-up showed good tolerability as no severe adverse events occurred, and all patients continued VIT treatment with maintenance administration every month.

The cohort of HVA patients showed a higher prevalence of Vespula venom sensitization in line with what has been reported in numerous studies [1,25,26]. This predominance is likely to be related to occupational factors and to the environment, mostly rural in southern Italy, where Messina is [27].

Other venom sensitization profiles included *Vespa crabro* and Apis mono-sensitization, and co-sensitization to Vespula and *Polistes dominula* in several patients.

*Vespa crabro* is usually reported in co-sensitization with Vespula [28], but in our HVA population, as in another population [29], mono-sensitization to *Vespa crabro* was present in all six patients. Stings from these Hymenoptera are quite easy to recognize as they are bigger than other wasps and are the only Hymenoptera in Europe that sting even at night [30].

Apis mono-sensitization in our population was present in beekeepers, and therefore, occupational exposure influenced sensitization [31].

As far as co-sensitization to *Vespula* and *Polistes dominula* in our population, it was only diagnosed through s-IgE determination, as IgE inhibition tests are not available in our allergy center; diagnostic strategies using cross-reactive major allergens are warranted [32,33]. Nevertheless, Alutard Vespula VIT proved to be effective even in *Polistes dominula* co-sensitized patients.

In our 5-year prospective observational study, all patients were at high risk for severe or potentially fatal anaphylaxis. Most of them were adult males with high occupational or recreational exposure to stinging insects, elements which are known to significantly influence both the likelihood of sting exposure and the clinical expression of the allergic response [34]. Two patients had a diagnosis of SM, a clinical condition known to be associated with an increased risk of SR to Hymenoptera stings [35].

VIT proved to be an effective treatment in our population of HVA patients, as no SR occurred after an occasional Hymenoptera sting while undergoing maintenance VIT, including SM patients. Therefore, our data confirms the life-saving characteristics of VIT [36] as the long-term observation period of 5 years allowed a comprehensive evaluation of the real-life protective efficacy of VIT. This finding represents a key clinical outcome of the study, as VIT demonstrated complete protection from SSR throughout the observational period.

The particularly favorable response observed in Vespula-sensitized individuals treated with Vespula VIT extract aligns with EAACI guidelines [1]. In general, VIT is reported to be effective in more than 90–95% of patients treated for wasp allergy and in 80–90% of patients allergic to bees [37]. This data may be correlated to Vespula venom, which is more stable and has less variable antigenic composition than Apis venom; the available commercial extracts are generally more complete and more standardized in terms of content of major allergens, such as Ves v 5 and Ves v 1 [38].

Our results document a progressive reduction in laboratory biomarkers such as s-IgE as well as of BST levels. Vespula-specific IgE presented a highly statistically significant decrease; Incorvaia et al. [39] also reported highly favorable responses to VIT in Vespula-sensitized individuals. Furthermore, in our study of poly-sensitized patients, both venom s-IgE to Vespula and *Polistes dominula* significantly decreased. This data is in line with what was reported by Blank et al. [40], who highlighted that VIT can consistently reduce total IgE and s-IgE serum levels also in poly-sensitized patients.

It has been reported that the risk of a life-threatening reaction after a *Vespa crabro* sting is higher compared with Apis or Vespula stings in Mediterranean countries [41]. The evidence of a statistical reduction in *Vespa crabro* s-IgE after 5 years of *Vespa crabro* Anallergo VIT treatment confirms its efficacy [42].

In our study, BST levels showed a reduction over the five years of VIT treatment, but not statistically significant. These results confirm that the effect of VIT is mainly on the decrease in s-IgE secondary to IgG4 increase [43], but it still suggests that VIT might decrease systemic mast cell activity over time. It has been reported that VIT, while not leading to a significant reduction in tryptase, can contribute to a greater stability of the immunological and clinical profile of treated patients, reducing susceptibility to SR [44]. VIT duration could be relevant to the reduction in BST levels, as in a study in children, BST levels did not change after 1 year of VIT [45].

Our data gives a comprehensive overview of immunological changes during VIT in a real-world setting. Clinical changes also occurred in 17/35 HVA patients, as occasional field stings caused, in all of them, clinical manifestations that were markedly attenuated compared to the grade of reaction experienced before starting VIT.

Clinical allergen tolerance depends on multiple mechanisms across different immune cell and tissue compartments, and therefore, more information must be acquired on how the immunological tolerance of VIT occurs [46].

### Study Limitations and Future Perspectives

The results of our study have limitations because of the restricted sample size, which prevents drawing firm conclusions. Nonetheless, the evaluation of the variation in clinical and immunological laboratory data in HVA patients from T0, before starting VIT, until T1, after 5-year VIT treatment, supports the evidence of VIT as a disease-modifying treatment. More studies on wider HVA populations are needed.

Further explorations, also considering Hymenoptera molecular allergens and the evolution of their levels undergoing VIT, could be of interest.

## 4. Conclusions

The data reported in this study strengthen the present evidence on VIT as a life-saving treatment, with the ability to induce significant immunological modulation even in poly-sensitized subjects. From a clinical and social point of view, VIT is confirmed as an intervention with a high preventive impact, capable of reducing morbidity, mortality and indirect costs related to the management of anaphylactic reactions from Hymenoptera stings in sensitized subjects. Its value relies not only on therapeutic efficacy, but also on restoring the perceived safety and quality of life of patients, often limited by the fear of occasional stings [47]. Looking ahead, the integration of risk stratification and personalized immunological monitoring is the key to further optimizing the selection of VIT candidates for Hymenoptera venom, modulating treatment duration and identifying subjects with suboptimal response at an early stage.

This real-life study confirms that VIT de-sensitizing treatment is a highly effective, safe and disease-modifying therapeutic strategy, capable of offering patients with HVA not only clinical protection, but a real restitutio ad integrum of their daily life.

## 5. Materials and Methods

### 5.1. Study Design and Clinical Setting

A prospective real-world study on HVA patients treated with VIT between May 2020 and December 2025 was carried out at the Allergy and Clinical Immunology Unit of the University Hospital of Messina, Messina, Italy. Ethical approval was obtained from the Ethics Committee of the University Hospital G. Martino of Messina University, at first on 27 April 2020 (Protocol number 24-20) and with a more specific profile on 2 April 2024 (Protocol number 34-24). Both ethical approvals, the 24-20 and 34-24, included collection of HVA patients’ characteristics, serum evaluation of Hymenoptera venom specific IgE and BST levels before starting Hymenoptera VIT as well as reporting tolerance to occasional Hymenoptera stinging during VIT; the 34-24 approval included the collection of Hymenoptera venom-specific IgE and BST levels before (T0) and after (T1) 5-year VIT treatment. All patients were included in the study under both approvals, and informed consent was obtained from all participants under both approvals; they were also informed about privacy and the use of anonymous data for research purposes and signed informed consent according to the European General Data Protection Regulation 2016/679.

### 5.2. Study Population

Subjects were enrolled if they had experienced a previous SSR to a Hymenoptera sting, classified according to Mueller’s grading system as grade III or IV reaction, showed clear evidence of IgE-mediated sensitization to Hymenoptera venom allergens, and started VIT. Only patients treated with VIT for five years were included, to ensure a reliable assessment of long-term clinical outcomes.

**Figure 2:**
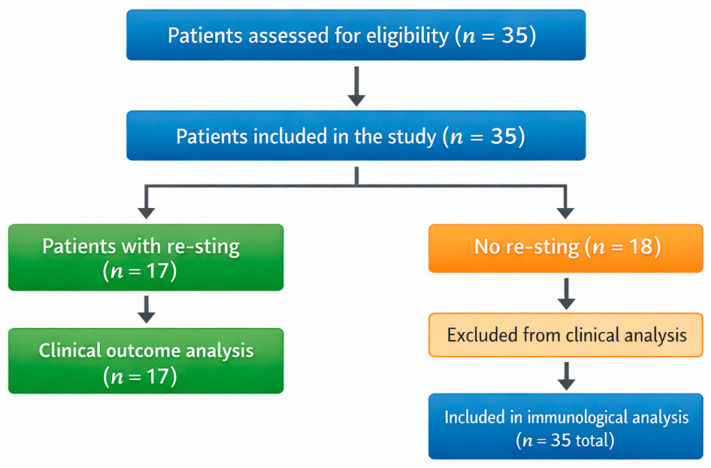
Flow diagram of patient selection and analysis. The flow diagram shows a total of 35 patients included in the study. Among them, 17 patients experienced at least one accidental re-sting during follow-up and were included in the clinical outcome analysis. The remaining 18 patients did not report any re-sting and were therefore excluded from clinical evaluation. However, all 35 patients were included in the immunological analysis, as laboratory parameters (total IgE, venom-specific IgE, and SBT) were available at both baseline (T0) and follow-up (T1).

Patients presenting exclusively local reactions or mild systemic reactions (Mueller grade I–II) after a Hymenoptera sting, as well as those with incomplete clinical or laboratory data or with contraindications to VIT, such as systemic autoimmune diseases or end-stage organ damage, were excluded from the analysis. A total of 35 patients were initially assessed and included in the study; no dropouts occurred, and all 35 patients were included in the final analysis population. During follow-up, only 17 patients experienced at least one accidental re-sting and were therefore eligible for clinical outcome evaluation. The remaining 18 patients did not experience any re-sting during the observation period and were consequently excluded from the clinical efficacy analysis. However, all 35 patients were included in the immunological analysis, as baseline (T0) and follow-up (T1) laboratory parameters—including total IgE, specific IgE to Hymenoptera venoms, and SBT—were available regardless of re-sting occurrence; this approach allowed for the separation between clinical effectiveness (restricted to re-sting patients) and immunological response (evaluated in the entire cohort) to avoid biases in clinical outcome assessment (Figure 2).

### 5.3. Clinical Evaluation

A comprehensive allergy history was collected from all patients. Emphasis was placed on the clinical description of the index sting reaction, including the type of symptoms, their time of onset, progression, and resolution. Information regarding previous sting events, occupational exposure, outdoor and recreational activities, and potential risk factors was also recorded. Whenever feasible, identification of the stinging insect was attempted based on the patient’s description.

### 5.4. Diagnostic Work-Up

The measurement of laboratory parameters such as total serum IgE and s-IgE to Apis, Vespula, *Polistes dominula* and *Vespa crabro* venoms, as well as BST levels (Pharmacia-Cap System, Stockholm, Sweden), was performed. These immunological markers were evaluated at two points: baseline (T0), before the initiation of VIT, and at the end of the 5-year maintenance treatment (T1).

### 5.5. VIT

All patients were treated with commercially available, standardized venom extracts, selected based on the identified sensitization profile: Alutard Apis and Alutard Vespula venom extracts (ALK-Abellò, Milan, Italy) and Anallergo *Vespa crabro* venom extracts (Anallergo, Scarperia e San Piero, Italy). All venom extracts used for VIT were depot extracts: Alutard Apis and Vespula extracts were adsorbed on aluminum hydroxide [48], while Anallergo *Vespa crabro* extract was adsorbed on L-Tyrosine [49]. The venom extracts used for VIT treatment were all commercially manufactured pharmaceutical products. VIT with all venom extracts was administered according to an accelerated build-up protocol [50] at a concentration of 100 mcg. The accelerated schedule consisted of four subcutaneous administrations of 0.01, 0.03, 0.06, and 0.15 mL (cumulative dose of 25 mcg) on the first day, whereas four other administrations, all at a dosage of 0.25 mL (cumulative dose of 100 mcg) on the second day. Each patient received VIT doses at 30 min intervals on the first and second day. Maintenance doses were administered first after 15 days and then monthly thereafter, with a cumulative dose of 100 mcg, in two consecutive subcutaneous injections of 50 mcg in each arm.

Therefore, the total number of procedures from T0 to T1 was 128 subcutaneous VIT injections, specifically 32 subcutaneous injections in the 1st year and 24 each year from the 2nd to the 5th year. The cumulative dose of venom extract administered to each patient from T0 to T1 was 6100 mcg of venom extract; 13,000 mcg in the first year and 4800 from the second to the fifth year, respectively.

Throughout both the induction and maintenance phases, patients were monitored for treatment tolerability.

### 5.6. Follow-Up and Clinical Outcome Assessment

All patients were followed for a period of up to five years after VIT initiation. During follow-up, particular attention was paid to the occurrence of accidental field re-stings, the type of reaction following re-sting (absence of reaction, local reaction, large local reaction or SR) and tolerance to VIT, recording eventual adverse events related to treatment.

### 5.7. Statistical Analysis

Data analysis was performed using descriptive and inferential statistical methods. Continuous variables were expressed as median values, whereas categorical variables were reported as absolute numbers and percentages. Longitudinal comparisons between baseline and follow-up values were carried out to assess temporal variations in immunological parameters. Statistical analysis was conducted by the Wilcoxon test for paired data and the Bonferroni correction for multiple comparisons (α = 0.0083). The level of statistical significance was set at *p*-value < 0.05.

## Figures and Tables

**Table 1:** Comparison of total IgE, venom s-IgE, and BST levels between baseline (T0) and follow-up (T1). Statistical significance was assessed using paired tests according to data distribution, with *p*-values < 0.05 considered statistically significant.

Marker	Paired Data	Wilcoxon *p*	Outcome
Total IgE	30	0.0028	Statistically significant reduction
Baseline serum tryptase	10	0.4922	Reduction, but not statistically significant
s-IgE Vespula	31	0.0003	Highly statistically significant reduction
s-IgE *Polistes dominula*	29	0.0177	Statistically significant reduction
s-IgE Apis	31	0.0133	Statistically significant reduction
s-IgE *Vespa crabro*	26	0.0222	Statistically significant reduction

## Data Availability

The datasets generated during the current study are not publicly available but could be made available from the corresponding author on reasonable request. The data is not publicly available due to regulatory and ethical restrictions.

## Supplementary Materials

The following supporting information can be downloaded at: https://www.mdpi.com/article/10.3390/toxins18040187/s1, Supplementary Table S1: Immunological parameters before (T0) and after (T1) VIT. Total IgE, BST, and venom-specific IgE (sIgE) to Apis mellifera, Vespula species, Vespa crabro, and Polistes dominula are reported for each patient at baseline (T0) and follow-up (T1). Total IgE is expressed as UI/mL, BST as ng/mL, and specific IgE as kUA/L.

## Abbreviations

The following abbreviations are used in this manuscript:

HVA	Hymenoptera venom allergy
VIT	Venom immunotherapy
SSR	Severe systemic reaction
SR	Systemic reaction
BST	Baseline serum tryptase
SM	Systemic mastocytosis
MCAS	Mast cell activation syndrome
