# Supplementary material for: Five-Year Real-World Outcomes of Hymenoptera Venom Immunotherapy: Clinical Effectiveness and Immunological Modifications
Source: Toxins (Basel). 2026 Apr 15;18(4):187. doi: 10.3390/toxins18040187 (PMC13120408; doi:10.3390/toxins18040187)
Supplement: Supplementary file 1 [file toxins-18-00187-s001.zip › toxins-4208325-supplementary.pdf]

Supplementary Materials: Five-Year Real-World Outcomes of Hymenoptera Venom Immunotherapy: Clinical Effectiveness and Immunological Modifications

Claudia Panzera, Sebastiano Gangemi and Luisa Ricciardi

Table S1. Immunological parameters before (T0) and after (T1) VIT. Total IgE, BST, and venom-specific IgE (sIgE) to Apis mellifera, Vespula species, Vespa crabro, and Polistes dominula are reported for each patient at baseline (T0) and follow-up (T1). Total IgE is expressed as UI/mL, BST as ng/mL, and specific IgE as kUA/L.

ID	Age	Sex	Venom	Mueller Grade	Total IgE T0 (UI/mL)	Total IgE T1 (UI/mL)	BST T0 (ng/mL)	BST T1 (ng/mL)	Apis T0 (kUA/L)	Apis T1 (kUA/L)	Vespa crabro T0	Vespa crabro T1	Polistes T0	Polistes T1	Vespula T0	Vespula T1
1	30	M	Vespula species	III	110.0	73.8	5.37	6.1	0.1	0.02	0.6	0.4	15.3	6.11	8.1	2.34
2	53	M	Vespula species	III	19.7	10.0	4.68	2.0	0.41	0.1	1.0	0.23	0.71	0.2	0.9	0.27
3	63	M	Vespa crabro	IV	84.4	49.6	7.33	8.5	0.25	0.12	1.29	0.16	2.13	0.4	18.6	1.27
4	31	M	Vespula species	IV	36.4	18.1	4.60	4.3	0.79	0.12	0.82	0.78	0.1	0.1	0.84	0.73
5	17	M	Apis mellifera	IV	945.0	836.0	2.8	2.5	5.11	1.88	0.9	0.7	0.1	0.17	0.51	0.46
6	62	M	Vespula species	III	535.0	321.0	3.8	4.0	1.3	0.9	5.54	3.68	21.2	6.91	26.0	17.5
7	35	M	Vespula species	III	224.0	121.0	3.2	3.0	0.22	0.6	3.5	1.53	1.3	0.86	18.0	8.38
8	47	F	Vespula species	III	44.9	144.0	11.5	10.0	0.1	0.09	14.9	2.61	5.34	1.65	14.6	4.14
9	30	F	Apis mellifera	IV	100.0	15.3	4.8	4.6	27.5	1.19	0.36	0.25	0.29	0.1	0.32	0.19
10	41	F	Apis mellifera	III	95.0	56.4	3.9	3.0	2.75	1.67	0.0	0.1	0.0	0.1	0.15	0.1
11	73	F	Apis mellifera	III	450.0	276.0	8.0	7.5	2.86	2.47	0.2	0.23	0.17	0.13	0.18	0.21
12	49	M	Vespula species	III	103.8	262.0	3.8	4.3	1.09	1.45	4.56	3.82	9.68	2.89	25.8	10.9
13	57	M	Vespa crabro	IV	52.7	29.7	4.16	3.7	0.1	0.02	4.49	0.2	1.67	0.87	6.09	3.1
14	42	M	Vespula species	IV	216.9	100.0	8.4	6.8	0.1	0.08	1.76	1.74	0.97	0.63	12.0	10.0
15	45	M	Vespa crabro	III	12.0	22.9	6.5	6.3	0.11	0.23	0.34	0.87	0.29	1.08	1.4	3.85

16	68	M	Vespula species	IV	34.6	23.3	23.3	34.6	0.29	0.12	0.48	0.48	0.65	0.15	1.26	0.7
17	45	M	Vespula species	IV	114.6	126.0	7.8	7.6	0.93	2.97	1.7	2.39	1.88	1.04	5.34	4.3
18	60	M	Vespula species	III	61.0	54.6	4.7	4.5	0.1	0.14	7.76	0.31	31.7	1.63	5.02	2.47
19	39	M	Vespula species	IV	76.3	51.5	5.06	5.7	0.21	0.11	1.0	0.11	1.01	0.47	73.5	3.4
20	39	M	Vespula species	III	597.0	122.0	5.0	6.0	10.1	0.37	21.5	2.53	1.33	1.62	28.4	5.31
21	25	M	Vespula species	III	100.0	15.3	4.65	3.7	0.45	0.36	5.3	4.61	23.0	14.0	45.3	8.93
22	17	M	Vespula species	III	259.0	196.0	5.20	4.61	0.1	0.1	0.63	0.22	0.42	1.06	3.08	1.34
23	44	M	Vespula species	IV	57.6	125.0	5.21	5.0	0.1	0.1	1.39	0.1	0.1	0.1	1.45	0.83
24	50	F	Vespula species	III	447.0	208.0	3.2	3.4	0.11	0.05	0.26	1.74	0.1	0.19	1.0	0.5
25	39	M	Vespula species	IV	33.6	122.0	52.0	51.2	0.1	0.1	0.1	0.1	0.1	0.1	0.39	0.2
26	46	F	Apis mellifera	IV	180.0	52.0	2.0	3.0	30.8	24.6	0.18	0.1	0.1	0.1	0.1	0.1
27	64	M	Vespula species	IV	37.3	37.8	8.6	9.7	0.1	0.0	0.1	0.19	0.1	0.05	2.96	0.18
28	44	M	Vespula species	IV	19.2	18.2	4.3	4.0	0.1	0.1	0.64	0.4	0.1	0.1	0.92	0.59
29	62	M	Vespa crabro	III	507.0	156.0	6.0	5.3	0.1	0.1	11.6	0.88	1.83	0.3	0.6	1.06
30	69	M	Apis mellifera	IV	202.0	96.0	6.4	6.1	52.8	9.11	1.47	5.05	0.32	2.51	2.2	11.5
31	52	M	Vespula species	IV	16.3	26.8	3.0	3.5	0.1	0.1	1.2	0.84	0.1	0.1	2.1	2.07
32	47	M	Vespula species	IV	153.0	90.6	6.46	4.7	0.1	0.17	0.82	0.84	2.04	0.76	5.11	1.48
33	48	M	Vespula species	IV	51.1	31.3	6.0	6.7	0.1	0.03	0.78	0.4	0.3	0.33	0.8	1.77
34	47	M	Vespula species	IV	5.86	6.32	3.4	4.5	0.1	0.1	0.51	0.44	0.76	0.49	4.75	3.97
35	7	M	Vespula species	IV	100.0	15.3	4.6	3.7	1.29	0.74	2.3	0.99	0.40	0.19	5.85	1.67
